# FoxO1 signaling plays a pivotal role in the cardiac telomere biology responses to calorie restriction

**DOI:** 10.1007/s11010-015-2615-8

**Published:** 2015-12-26

**Authors:** N. Makino, J. Oyama, T. Maeda, M. Koyanagi, Y. Higuchi, I. Shimokawa, N. Mori, T. Furuyama

**Affiliations:** Division of Molecular and Clinical Gerontology, Department of Molecular and Cellular Biology, Medical Institute of Bioregulation, Kyushu University, 4546 Tsurumihara, Beppu, 874-0838 Japan; Department of Investigative Pathology, Nagasaki University School of Medicine, Nagasaki, Japan; Department of Anatomy and Neurobiology, Nagasaki University School of Medicine, Nagasaki, Japan; Department of Liberal Arts and Sciences, Kagawa Prefectural College of Health Sciences, Kagawa, Japan

**Keywords:** Calorie restriction, FoxO1, Telomere, Telomerase, Autophagy, Oxidative stress

## Abstract

This study examined whether the forkhead transcription factors of O group 1 (FoxO1) might be involved in telomere biology during calorie restriction (CR). We used FoxO1-knockout heterozygous mice (FoxO1^+/−^) and wild-type mice (WT) as a control. Both WT and FoxO1^+/−^ were subjected to ad libitum (AL) feeding or 30 % CR compared to AL for 20 weeks from 15 weeks of age. The heart-to-body weight ratio, blood glucose, and serum lipid profiles were not different among all groups of mice at the end of the study. Telomere size was significantly lower in the FoxO1^+/−^-AL than the WT-AL, and telomere attrition was not observed in either WT-CR or FoxO1^+/−^-CR. Telomerase activity was elevated in the heart and liver of WT-CR, but not in those of FoxO1^+/−^-CR. The phosphorylation of Akt was inhibited and Sirt 1 was activated in heart tissues of WT-CR and FoxO1^+/−^-CR. However, the ratio of conjugated to cytosolic light chain 3 increased and the level of p62 decreased in WT-CR, but not in FoxO1^+/−^-CR. A marker of oxidative DNA damage, 8-OhdG, was significantly lower in WT-CR only. The level of MnSOD and eNOS increased, and the level of cleaved caspase-3 decreased in WT-CR, but not FoxO1^+/−^-CR. Echocardiography showed that the left ventricular end-diastolic and systolic dimensions were significantly lower in WT-CR or FoxO1^+/−^-CR than WT-AL or FoxO1^+/−^-AL, respectively. The present studies suggest that FoxO1 plays beneficial roles by inducing genes involved in telomerase activity, as well as anti-oxidant, autophagic, and anti-apoptotic genes under conditions of CR, and suggest that FoxO1 signaling may be an important mediator of metabolic equilibrium during CR.

## Introduction

Both clinical and experimental studies have shown that calorie restriction (CR) is capable of extending life span and lowering the onset of chronic diseases as well as overall disease morbidity and mortality [1, 2]. CR has been shown to exert some profound cardiovascular effects, such as lowering blood pressure [3], decreasing systemic inflammation [4], and improving cardiac diastolic parameters [5]. The exact mechanisms by which CR exerts these cardiovascular effects remain largely elusive, although the most prevalent theory points to a significant protection from DNA damage due to a reduction of metabolism [6, 7].

Understanding the mechanisms underlying caloric restriction is of great importance as this could pinpoint new therapeutic targets for age-associated diseases, or for anti-aging therapies. In this regard, the well-documented association between telomere shortening and aging [8] suggests a possible role of telomere dynamics in the systemic effects of caloric restriction. The length of leukocyte telomeres is inversely related to the body mass index and insulin resistance [9], and shortened leukocyte telomeres are associated with various age-related diseases such as atherosclerosis [10]. Although emerging evidence indicates that both the telomeres and telomerase activity control key cellular functions, including replicative lifespan, cell differentiation and cell proliferation, the molecular basis of these effects, and their relations to the presumed cardiac signals for the forkhead transcription factors of O group (FOXO), remain unknown.

Studies on the mechanisms of caloric restriction-related longevity in budding yeast have identified the silent information regulator 2 (Sir2) as a survival factor that prolongs lifespan [11, 12]. Sirt1, a mammalian homolog of Sir2, was originally identified as an NAD-dependent histone deacetylase [13]. Recent studies have shown that Sirt1 is involved in the regulation of a wide variety of cellular processes, ranging from stress response, cell cycle, metabolism, and apoptosis in response to the cellular energy and redox status, through its deacetylase activity [14]. The forkhead transcription factors of the O group (FoxO) are among the well-documented targets of Sirt1 in cardiomyocytes. One member of this family, FoxO1, plays important roles in systemic homeostasis, among other biological functions [15]. In mice, the loss of FoxO1 is embryonically lethal, whereas FoxO3 deletion results in normal birth but the offspring are prone to cardiac hypertrophy and eventual cardiac failure [16]. FoxO1 is involved in regulating various cellular processes in different tissues, including the oxidative stress response, cell proliferation, immune homeostasis, pluripotency in embryonic stem cells, cell death, and metabolism [15]. During the oxidative stress response, FoxO1 is known to increase the expression of such anti-oxidant genes as superoxide dismutase, thereby promoting reactive-oxygen-species (ROS) scavenging activity, preventing DNA damage, and fundamentally safeguarding cells from damage [17]. FoxO1 also plays an important role in cell-longevity through its collaborative activity with Sirt1, which itself turns on the transcription of anti-oxidant genes such as MnSOD and catalase [17].

Autophagy is a self-digestion process through which cells degrade their own components, thereby redirecting amino acids, fatty acids, and carbohydrates to energy production or synthesis of essential cellular molecules [18]. The autophagic mechanism for recycling the cellular building blocks plays an important role during CR. In addition, autophagy is induced during increased ROS generation. Indeed, it has been proposed that upregulation of autophagy is a major mechanism underlying the lifespan-extending properties of CR [19].

To investigate the potential role of FoxO1 in the cardiac effects of caloric restriction, FoxO1-knockout heterozygous mice (FoxO1^+/−^), in which the level of Foxo1 mRNA was reduced by 50 % or more [20], were used in the present study. Both wild-type (WT) and FoxO1^+/−^ mice were subjected to either 30 % caloric restriction (CR; feeding with 70 % of the control diet) or ad libitum feeding (AL) for 20 weeks. We assessed the telomere biology, including the telomere length and telomerase activity, and the essential signaling pathways responsible for cell survival including autophagy, and cardiac geometry. In this way, we sought to clarify whether FoxO1 signaling is an important mediator of the maintenance of telomere biology in response to CR.

## Methods

### Experimental animals

**Fig. 1:**
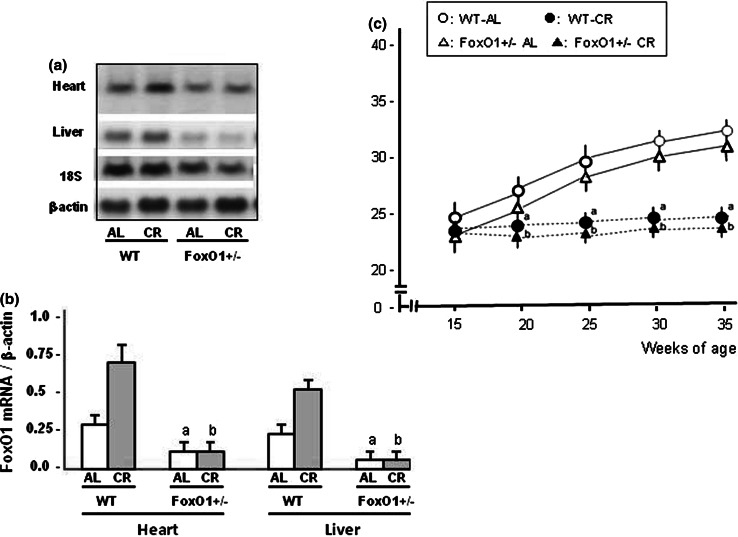
FoxO1 mRNA expressions and body weight changes in wild-type (WT) and FoxO1^+/−^ mice fed ad libitum (AL) or subjected to calorie restriction (CR). Representative data for the mRNA expressions of FoxO1 in the heart and liver tissues are shown in (**A)** and summarized results are shown in (**B**). Body weight was measured in four experimental groups in (**C**). *Values* are the mean ± SE (*n* = 6 animals in each group). **a** *p* < 0.05 versus WT-AL at the same age. **b** *p* < 0.05 versus FoxO1^+/−^-AL at the same age

Homozygous FoxO1-knockout mice are embryonic lethal (20), and thus heterozygous FoxO1-knockout mice (FoxO1^+/−^) were used for the present experiments. The level of *Foxo1* mRNA in the FoxO1^+/−^ was reduced by 50 % or more in the liver and heart (Fig. 1A, B). Wild-type mice (WT) (C57BL/6J) were used as a control. FoxO1^+/−^ mice were generated and backcrossed onto a C57BL/6J background at the National Institute of Longevity Sciences (Obu, Aichi, Japan) and were transferred to the Animal Center at the Kyushu University Beppu Hospital. Tail biopsies of FoxO1^+/−^ and WT mice were performed in weanling mice for genotyping by PCR with specific primers. Mice were housed individually in plastic cages (one animal/cage) in a barrier facility (temperature, 22 ± 1.0 °C; 12 h light/dark cycle) under specific pathogen-free conditions that were maintained for the entire study. All animal experiments conformed to *the Guide for the Use and Care of Laboratory Animals* (NIH Pub. No. 85-23, revised 1996) issued by the U.S. National Institutes of Health, and approved by the Kyushu University Institutional Animal Care and Use Committee.

### CR diet

Each mouse was individually housed until 8 weeks of age. The average caloric intake was calculated from the daily food intake over 2 weeks. At 12 weeks of age, the WT group and the FoxO1^+/−^ mice group were each randomly divided into two groups, an ad libitum (AL) group and a CR group. WT-AL mice were fed AL until the end of study, whereas the WT-CR mice were subjected to restriction of the average AL caloric intake for 3 weeks (10 % restriction for acclimation) followed by a 30 % caloric reduction from 15 to 35 weeks of age (Fig. 1B). The CR diet was enriched in vitamins and minerals to ensure constant daily intake of those nutrients. All mice were fed AL with a Charles River-LPF diet (Oriental Yeast Co., Ltd., Tsukuba, Japan) as a standard diet for long-term studies, including studies of the CR regimens in mice. Body weight was monitored every week from 15 to 35 weeks of age. Insulin resistance was evaluated using the value of homeostasis model assessment of insulin resistance (HOMA-IR) as a marker [21, 22]. HOMA-IR was determined based on both plasma glucose and serum insulin levels. At 35 weeks of age, the mice were decapitated, and the heart and liver tissues were collected for the following analyses. These tissues were immediately frozen in liquid nitrogen and stored at −80 °C until assayed.

### Extraction of genomic DNA from mouse tissues

Mouse tissue samples were lysed by incubation at 55 °C for 48 h in 200 μL lysis buffer containing 10 mM Tris/HCl (pH 8.0), 0.1 mM EDTA (pH 8.0), 2 % sodium dodecyl sulfate (SDS), and 500 μg/mL protease K (Roche Diagnostic, Tokyo, Japan). Genomic DNA extraction was performed using a DNeasy Tissue Kit (Qiagen K.K.,Tokyo, Japan) according to the manufacturer’s recommendations, as described previously [22].

### Measurement of telomere length and telomerase activity

The length of the telomere DNA was estimated as the telomere-to-centromeric DNA content ratio, as previously reported [23]. Telomerase activity was examined using a modified telomerase repeat amplification protocol (TRAP) assay [23] with TeloChaser (Toyobo, Osaka, Japan) according to the manufacturer’s instructions. The intensities of the bands were quantified with ImageJ (NIH). For each genotype, telomerase activity was analyzed in seven types of tissues from 3 to 6 animals. The assays were repeated at least twice for each animal in order to ensure reproducibility. A human cancer cell line overexpressing telomerase was used as a reference in each assay.

### Immunohistochemistry

For histological analysis, heart tissues (*n* = 6 in each group) were immersed in 10 % buffered formalin. The fixed tissues were dehydrated, embedded in paraffin, sectioned into 4 μm slices and stained with hematoxylin and eosin. As a marker of oxidant stress, the level of 8-hydroxydeoxyguanosine (8-OHdG) was quantified using a previously reported procedure [22]. To evaluate autophagy, immunofluorescence staining for LC3 was detected in heart tissue using a primary antibody to rat LC3 (No.010-22841,1:400) [23].

### Western blotting

Total protein was extracted from frozen hearts. Equal amounts of total protein (20–40 mg) were subjected to SDS-PAGE [23]. Left ventricular tissue was homogenized with 500 μL lysis buffer (100 mM Tris, pH 6.8, 4 % SDS, 20 % glycerol) containing the following protease inhibitors: 0.1 mM phenylmethanesulfonyl fluoride, 0.5 μL leupeptin, and 0.5 μL aprotinin. The Western blot analyses were carried out using the methods described in our previous reports [22, 23].

### Echocardiography

Transthoracic echocardiograms were recorded in conscious, sedated mice as described previously [23]. In brief, views were taken in planes that approximated the parasternal short-axis view (chordal level) and the apical long-axis view in the mice. The left ventricular (LV) internal diameters and wall thicknesses were measured (over at least 3 cardiac cycles) at end-systole and end-diastole. Fractional shortening (FS) was determined by the following equation: FS = [(left ventricular end-diastolic dimension (LVEDD) − left ventricular end-systolic dimension(LVESD))/LVEDD] × 100. The transmitral flow velocity profile was determined by positioning a sample volume at the tip of the mitral valve on the apical 4-chamber view. The peak velocity (E), the late velocity (A) and the deceleration time (DT) of the early diastolic filling wave were measured.

### Statistical analysis

Data are presented as the mean ± SEM. For intergroup comparisons, data were analyzed by one-way ANOVA, followed by Student’s *t* tests for unpaired data with Bonferroni’s correction. *p* < 0.05 was considered significant.

## Results

### Animal characteristics

Figure 1A shows the mRNA expressions of FoxO1 in the heart and liver tissues of experimental mice. The levels of FoxO1 mRNA expression were lower in the heart and liver of FoxO1^+/−^ -AL mice than in those of WT-AL mice by 30 and 10 %, respectively (Fig. 1B). The body weight gradually increased in both WT-AL and FoxO1^+/−^-AL mice independent of weeks of age until the end of the study (Fig. 1C). There was no significant difference in body weight between WT-AL and FoxO1^+/−^-AL or between WT-CR and FoxO1^+/−^-CR. The mean daily food intake in the FoxO1^+/−^-AL mice was not significantly different from that in WT-AL mice, and was also not significantly different between WT-CR and FoxO1^+/−^-CR mice during the study. CR abolished the significant increase in body weight for both the WT and FoxO1^+/−^. Table 1 shows the general profiles and echocardiographic data in the experimental mice at the end of the study. CR resulted in a smaller heart weight in WT-CR or FoxO1^+/−^-CR compared to WT-AL or FoxO1^+/−^-Al, respectively. However, the heart weight was not significantly different among all experimental mice. Thus, caloric-restricted mice displayed smaller body and heart weights without change in the ratio of heart weight to body weight. Although CR did not affect the fasting blood glucose and serum cholesterol levels in either WT or FoxO1^+/−^, the serum levels of insulin, HOMA-IR, and triglyceride were significantly lower in the CR groups than in the AL groups for both WT and FoxO1^+/−^ mice. Echocardiographic assessment revealed that both LVEDD and LVESD were significantly lower in WT-CR or FoxO1^+/−^ -CR than WT-AL or FoxO1^+/−^ -AL, respectively. The fractional shortening (FS) was not significantly different among the four groups. The interventricular septal wall thickness (IVST) and posterior wall thickness (PWT) were significantly reduced in WT-CR compared with WT-AL, but those changes were not observed in FoxO1^+/−^ mice. Markers of the diastolic function, the ratio of peak to late velocity (E/A) and the deceleration time (DT) of the mitral valve were not different among the four experimental groups.

**Table 1:** General characteristics and echocardiographic data in wild-type (WT) and FoxO1 knockout heterozygous mice (FoxO1^+/−^) fed with ad libitum (AL) or calorie restriction (CR)

	WT-AL (*n* = 8)	WT-CR (*n* = 8)	FoxO1^+/−^-AL (*n* = 6)	FoxO1^+/−^-CR (*n* = 6)
Body wt.(g)	35 ± 2.2	26 ± 1.7^a^	34 ± 1.4	25 ± .1.6^a,b^
Heart wt. (m g)	156 ± 7	117 ± 5^a^	146 ± 5	110 ± 4^a,b^
Heart to body wt (mg/g)	4.43 ± 0.27	4.50 ± 0.24	4.31 ± 0.26	4.40 ± 0.21
Fasting blood glucose (mg/dl)	96 ± 4.8	101 ± 6.8	105 ± 4.1	98 ± 4.8
Serum insulin (ng/ml)	0.41 ± 0.07	0.23 ± 0.05^a^	0.37 ± 0.04^a^	0.21 ± 0.04^a,b^
HOMA-IR	3.0 ± 0.16	1.5 ± 06^a^	2.8 ± .14	1.4 ± 0.07^a,b^
Total Cholesterol (mg/dl)	126 ± 6	118 ± 7	119 ± 5	116 ± 6
Triglyceride (mg/dl)	74 ± 2.1	62 ± 3.0^a^	78 ± 3.6	64 ± 3.8^a,b^
HR (bpm)	615 ± 30	585 ± 26	540 ± 25^a^	564 ± 31^a^
LVEDD (mm)	2.53 ± 0.08	2.26 ± 0.12^a^	2.47 ± 0.14	2.21 ± 0.13^b^
LVESD (mm)	1.25 ± 0.05	1.14 ± 0.04^a^	1.31 ± 0.04	1.17 ± 0.06^b^
IVST (mm)	1.18 ± 0.03	1.09 ± 0.04^a^	1.12 ± 0.03	1.08 ± 0.03
PWT (mm)	1.17 ± 0.06	1.02 ± 0.02^a^	1.13 ± 0.04	1.10 ± 0.03
E/A	2.5 ± 0.18	2.4 ± 0.17	2.4 ± 0.16	2.4 ± 0.16
DT (ms)	34 ± 1.2	35 ± 1.7	33 ± 1.7	34 ± 1.4
%FS	50 ± 1.7	50 ± 2.6	47 ± 2.4	46 ± 1.8

*HOMA*-*IR* homeostasis model assessment of insulin resistance, *HR* heart rate, *IVS* interventricular septal thickness, *LVPW* posterior wall thickness. *LVEDD* left ventricular end-diastolic diameter, *LVESD* left ventricular end-systolic diameter, %*FS* %fractional shortening, *E/A* the ratio of peak to late velocity, *DT* deceleration time

^a^ *p* < 0.05 versus WT-AL

^b^ *p* < 0.05 versus FoxO1^+/−^-AL

### CR and telomere biology

**Fig. 2:**
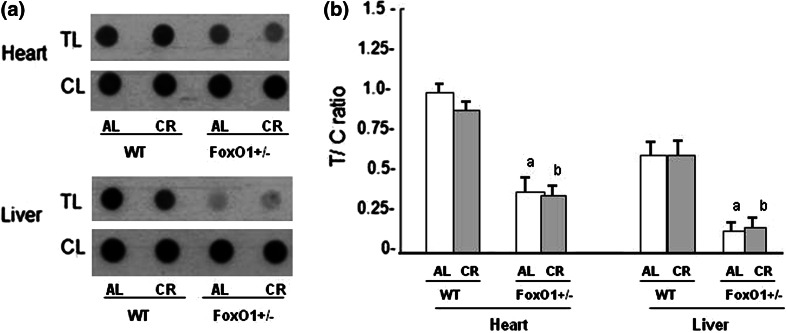
Dot blots of telomere length in heart and liver tissues from WT and FoxO1^+/−^ mice fed AL or subjected to CR. The length of telomere DNA, as assessed by dot-blot analysis, is presented as the telomeric-to-centromeric DNA content (T/C) ratio (**A**). Data were obtained from heart and liver tissues of each experimental mouse (**B**). The open column indicates mice fed an AL diet; the *gray column* indicates mice fed a CR diet. The *values* are the mean ± SE of six experiments. **a** *p* < 0.05 versus WT-AL

**Fig. 3:**
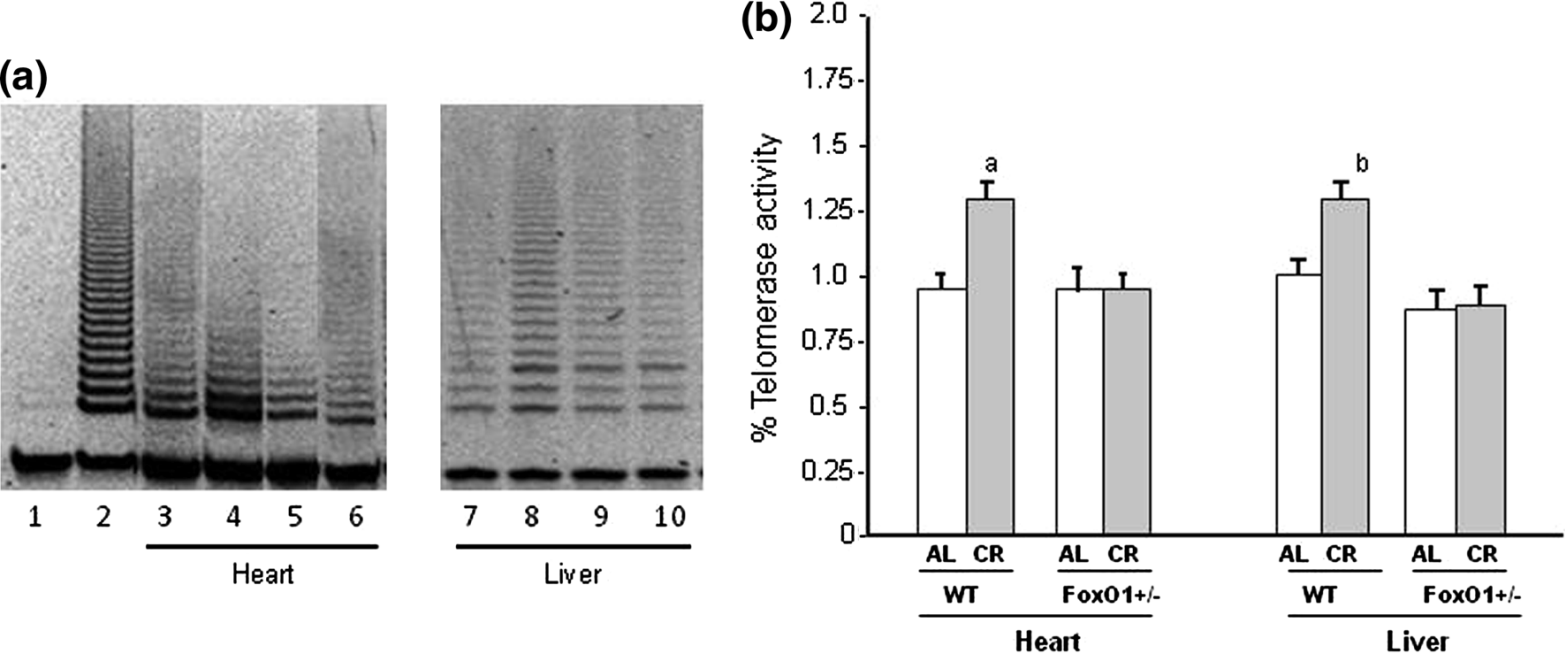
Telomerase activity as assessed by the telomerase repeat amplification protocol in heart and liver tissues from WT and FoxO1^+/−^ mice fed AL or subjected to CR. Representative data are shown in (**A**) and summarized results are shown in (**B**). *Data* are presented from the heart and liver tissues in each mouse. *Lane 1* negative control, *Lane 2* positive control, *Lanes 3* and *7* WT-AL, *Lanes 4* and *8* WT-CR, *Lanes 5* and *9* FoxO1^+/−^ -AL, *Lanes 6* and *10* FoxO1^+/−^-CR. *Values* are the mean ± SE of six experiments. **a** *p* < 0.05 versus WT-AL

**Fig. 4:**
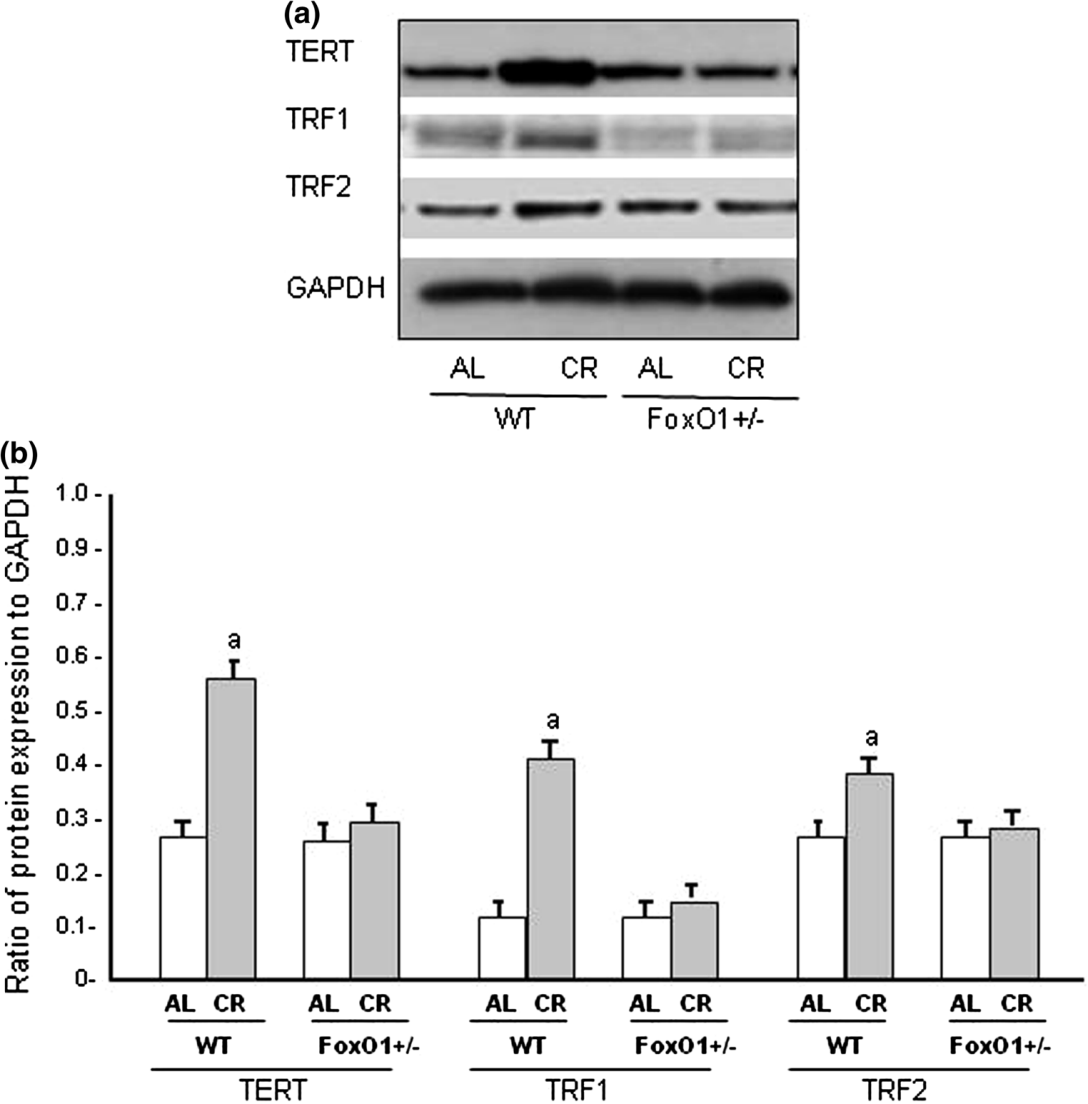
Protein expression levels of telomere reverse transcriptase (TERT), telomere repeat binding factor (TRF)1 and TRF2 in heart tissues from WT and FoxO1^+/−^ mice fed AL or subjected to CR. Representative data (**A**) and summarized results (**B**). Each group contained five animals. *Values* are the mean ± SE. **a** *p* < 0.05 versus WT-AL

The effect of partial loss of FoxO1 on the telomere DNA length was assessed with a dot-blot analysis using heart and liver specimens of mice at the end of the study (Fig. 2A). The telomere DNA length, as evaluated by the ratio of the density of telomeres to that of centromeres, was significantly shorter in both the heart and liver of FoxO1^+/−^ mice than WT mice regardless of CR. After CR, telomere attrition was not observed between AL and CR for either the WT or FoxO1^+/−^ mice. It was noticed that the telomere DNA length in the liver was shorter than that seen in the heart tissues (Fig. 2B). To determine whether the partial loss of FoxO1 affected telomerase function, we quantified telomerase activity using a TRAP assay. The level of telomerase activity of WT-CR was significantly higher than that of the WT-AL in both the heart and liver tissues (Fig. 3). Such an increase in telomerase activity by CR was not observed in FoxO1^+/−^ mice (Fig. 3). To obtain support for the CR-induced increase in telomerase activity, the expressions of the catalytic subunit TERT and telomere repeat binding factors 1 and 2 (TRF1 and TRF2) in the heart were assessed by Western blotting (Fig. 4). In line with the increase in telomerase activity, the level of TERT protein expression increased after CR in WT mice, and the expressions for TRF1 and TRF2 were also significantly increased in WT-CR. These changes in the TERT, TRF1, and TRF2 expressions were not observed in FoxO1^+/−^ mice. The results therefore suggested that CR activated telomerase in the heart tissues of the WT mice, but these affects were abolished in FoxO1^+/−^ mice.

### CR and expressions for Akt, Sirt 1, FoxO1, and FoxO3

**Fig. 5:**
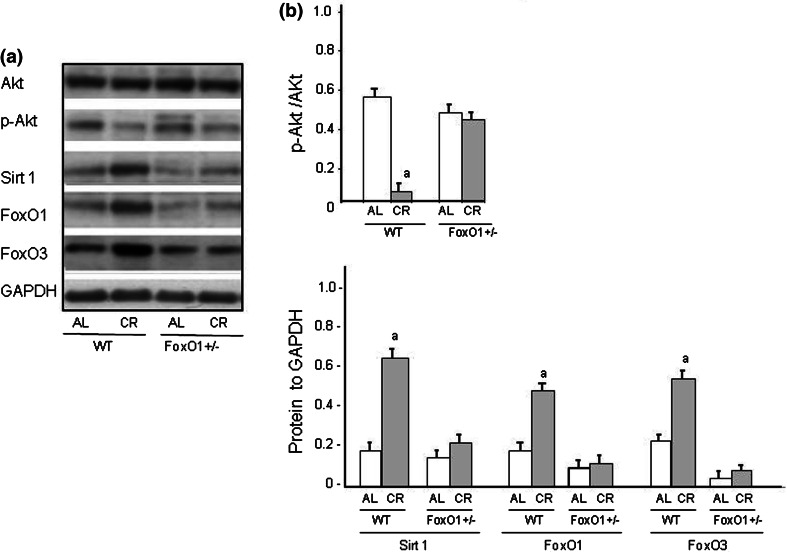
Western blot analyses of p-Akt, eNOS, FoxO1, FoxO3, and Sirt 1 in heart tissues from WT and FoxO1^+/−^ mice fed an AL or subjected to CR are shown in(**A**) and summarized results (*n* = 5) in (**B**). *Values* are the mean ± SE of six experiments. **a** *p* < 0.05 versus WT-AL. **b** *p* < 0.05 versus FoxO1^+/−^-AL

To obtain some mechanistic insights into the CR-induced increase in telomerase activity, the effects of CR-induced partial loss of FoxO1 expression on the level of protein expressions of Akt, Sirt 1, FoxO1, and FoxO3 as well as the level of Akt phosphorylation were evaluated in the heart (Fig. 5). p-Akt and Sirt 1 are known to be involved in the regulation of FoxO1 [24]. The CR significantly decreased the level of Akt phosphorylation in WT-CR mice, while there was no significant difference in FoxO1^+/−^ mice under either the AL or CR condition. In contrast, the CR significantly upregulated the level of Sirt 1 protein expression in WT-CR, but was not in FoxO1^+/−^. Furthermore, the CR significantly increased the expression of both FoxO1 and FoxO3 in WT, however, those expressions for FoxO1 and FoxO3 were not observed in FoxO1^+/−^ mice.

### CR and oxidative DNA damage and autophagy

**Fig. 6:**
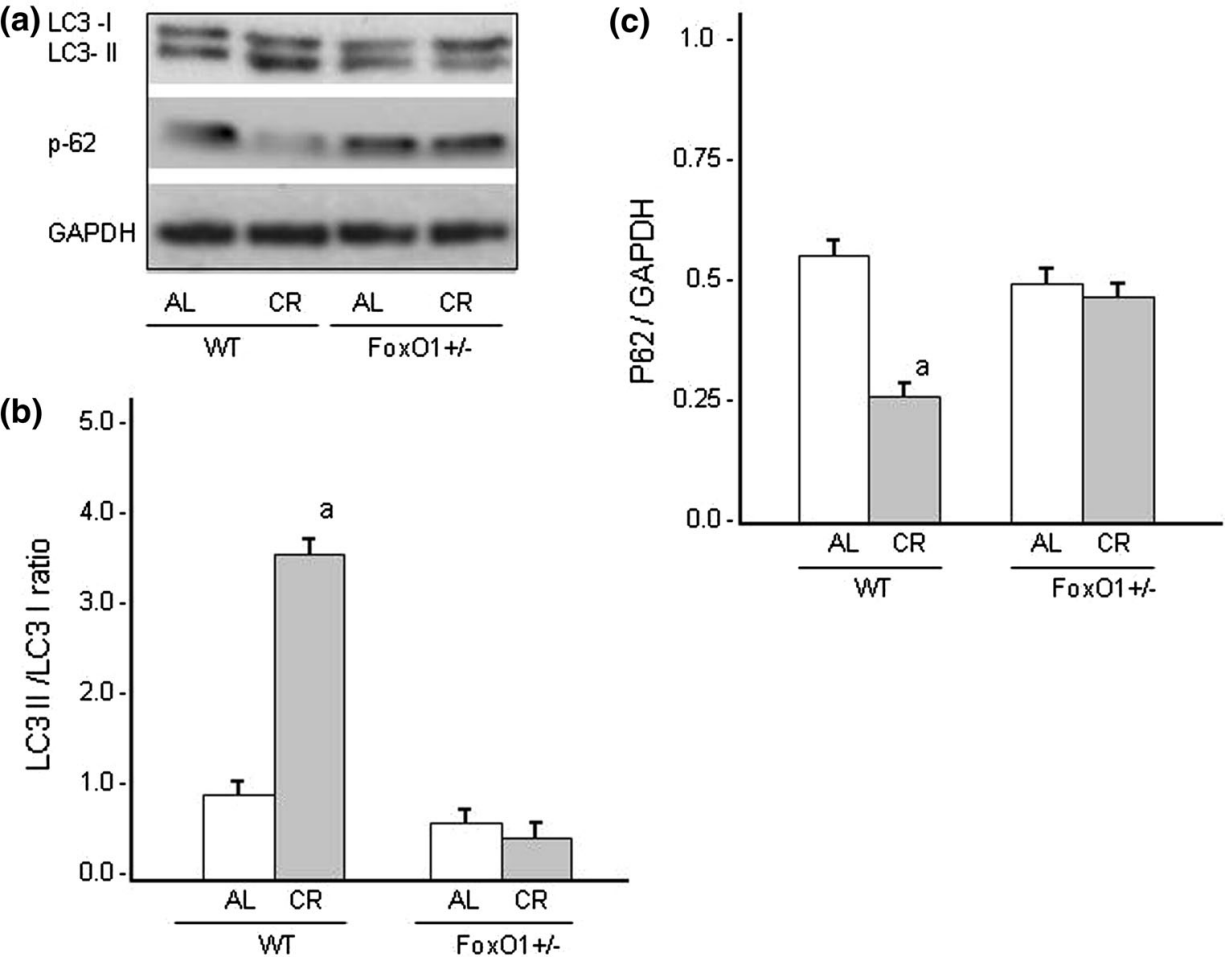
Western blot analyses of the levels of LC3 and p62 signaling in the hearts of WT and FoxO1^+/−^ mice fed an AL or subjected to CR. Representative data for the Western blots are shown in (**A**). Summarized results for the ratio of LC3II to LC3I are shown (**B**) and the protein expression for p62 is shown (**C**). *Values* are the mean ± SE of five experiments. **a** *p* < 0.05 versus WT-AL

**Fig. 7:**
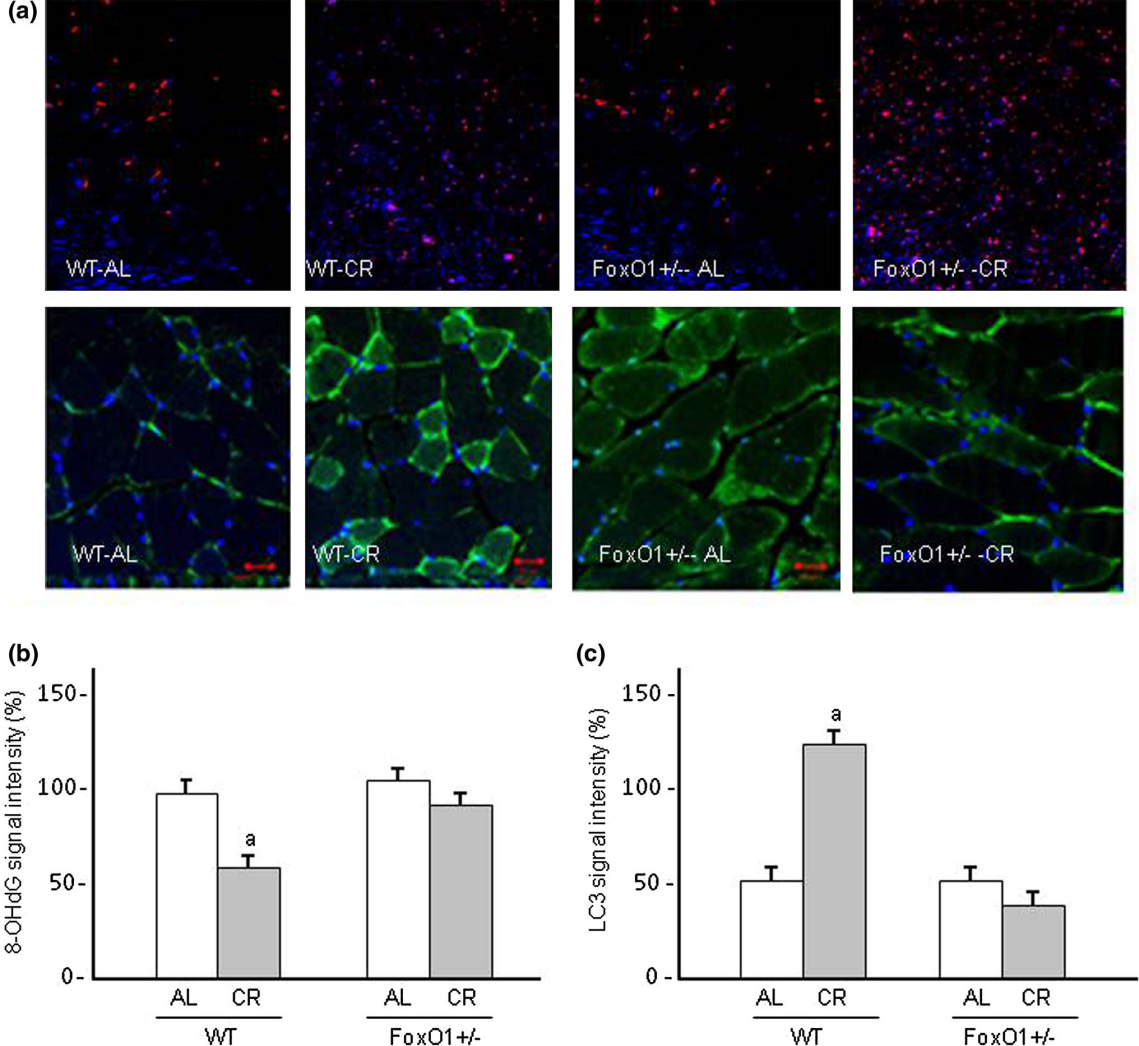
Immunohistochemical analyses of the levels of 8-OHdG and LC3 in the hearts of WT and FoxO1^+/−^ mice fed an AL or subjected to CR are shown (**A**) and the summarized results (*n* = 5) in (**B**). The upper portion of panel A shows the LV cross-sectional views of 8-OHdG staining (*red*) as a marker of oxidant stress in nuclei (**a**). Original magnification ×400. The lower portion in **A** shows LC3 staining with *green signals* indicating LC3 deposition. Original magnification, ×630. DAPI staining was performed as counterstaining. Summarized results are shown for 8-OHdG staining (**B**) and LC3 signal intensity (**C**). *Values* are the mean ± SE of five experiments. **a** *p* < 0.05 versus WT-AL. (Color figure online)

Conversion of LC3-I to LC3-II is an indicator of autophagosome formation, and p62 is an autophagy-specific substrate and degraded during autophagy [19]. It is therefore believed that the decrease in p62 abundance indicates autophagic activity. The effects of CR in FoxO1^+/−^ on the ratio of LC3-II to the cytosolic form of LC3 (LC3-I), and the level of p62 expression were evaluated in the heart (Fig. 6). The LC3-II/LC3-I ratio increased and the level of p62 expressions significantly decreased after CR in WT. However, these changes in the LC3-II/LC3-I ratio and p62 expression were not observed in FoxO1^+/−^ mice. In addition, immunofluorescence staining showed an increase in the LC3 level after CR, and this increase was absent in FoxO1^+/−^ (Fig. 7A, C). The present results therefore suggested that CR accelerated autophagy in the WT-CR mice, but not in the FoxO1^+/−^-CR animals. To investigate whether FoxO1^+/−^ mice had greater oxidative DNA damage in the heart, immunofluorescence staining of 8-OHdG (a marker of oxidative DNA damage) was performed (Fig. 7A). When the fluorescence intensities were quantitatively evaluated, the CR significantly decreased the level of 8-OHdG in WT-CR mice (Fig. 7B). However, these effects of CR were not observed in FoxO1^+/−^ mice-CR.

### CR and the expressions of MnSOD, caspase-3, and eNOS

**Fig. 8:**
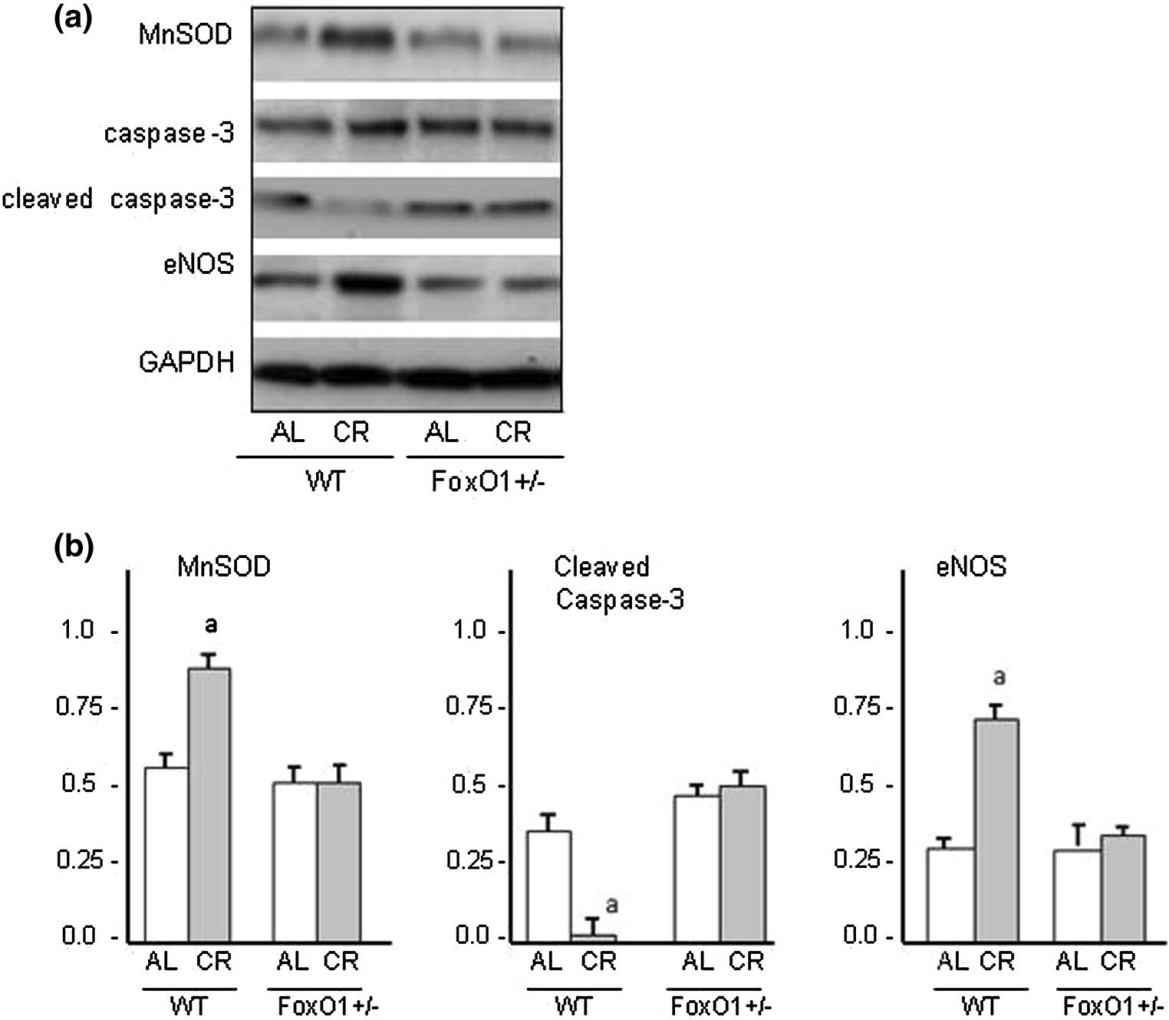
Western blot analyses of the levels of MnSOD, caspase-3, cleaved caspase-3, and eNOS in the hearts of WT and FoxO1^+/−^ mice fed an AL or subjected to CR and a summary of the results (**B**). *Values* are the mean ± SE of five experiments. **a** *p* < 0.05 versus WT-AL

FoxO1 is known to play beneficial roles in response to CR through the induction of anti-oxidant, autophagic, and anti-apoptotic genes [24, 25]. We have evaluated the protein expressions for MnSOD, cleaved caspase-3, and eNOS in the heart tissues of experimental mice. CR significantly increased the level of MnSOD and eNOS, and decreased the level of cleaved caspase-3 in WT mice (Fig. 8). However, these changes induced by CR were not observed in FoxO1^+/−^. Thus, CR-induced the activation of FoxO1, which in turn synthesized anti-oxidants such as MnSOD, thereby promoting cellular resistance against oxidative stress as well as the inhibition of apoptosis.

## Discussion

The present study demonstrated that the partial loss of FoxO1 in mice did not response to a CR-specific reaction, including of telomerase activity, autophagy, oxidant stress, and apoptosis. Morphological changes consisting of reductions of both LVEDD and LVESD were observed in WT-CR and FoxO1^+/−^-CR mice, but FS was not changed. The body weight and food intake were similar in WT and FoxO1^+/−^ mice under either ad libitum feeding or CR. CR decreased the level of serum insulin and improved the HOMA-IR index in both WT and FoxO1^+/−^ mice, although the glucose profiles and feeding conditions were similar to those mice. Thus, it seems that a partial loss of FoxO1 affects insulin resistance and altered energy metabolism, particularly under CR.

The telomeres were shorter in the heart tissues of FoxO1^+/−^-AL than in those of WT-AL, and the telomere attrition was not found to be a response to CR in either WT or FoxO1^+/−^. Our results showed that 20-week CR had no direct effect on telomere length in either the heart or liver tissues. It is possible that the duration of CR was insufficient and a longer period would be necessary for CR to exert its effect on the telomere length [2, 19]. Alternatively, it appears that the rate of telomere attrition is not consistent across organs in aged animals, as telomere in somatic cells reflects replicative history and predicts remaining proliferative potential [10, 25]. Thus, although the rate of telomere attrition in each organ may be age-dependent, cardiomyocytes are not originally proliferative and are terminally differentiated. These facts may support our finding that no telomere attrition occurred in response to CR. The telomerase activity was higher in the WT-CR than the WT-AL mice in this study, but this difference was not observed between the FoxO1^+/−^-CR and FoxO1^+/−^-AL. Thus, the present study suggests that FoxO1 signals may affect the telomere biology in the heart and liver tissues. It is also known that FoxO1 is beneficial qualities by highlighting their role in inducing anti-oxidant, autophagic, and anti-apoptotic genes under conditions of CR [17, 24]. FoxO1 also plays an important role in cell-longevity through the collaborative effect of FoxO1 with Sirt1, which turns on anti-oxidant genes such as MnSOD and catalase [24, 26]. The fact that FoxO1 reduced the load of telomere fragility may suggest a reduced replicative stress associated with CR in vivo, in agreement with the lower cellular proliferation described for this condition [27]. Alternatively, the observed telomere protection associated with CR could also be explained by the reduction of oxidative stress mediated by CR. In fact, oxidative stress accelerates telomere loss, whereas anti-oxidants decelerate it [28]. Our observations were confirmed to the FoxO1 signal may be associated with telomerase activity in heart tissues. With respect to telomerase, several studies have indicated that mice lacking telomerase function developed cardiac abnormalities, including dilated cardiomyopathy and reduced angiogenic potential [25]. In contrast, forced telomerase expression has been shown to lead to prolonged cardiomyocyte cycling and hypertrophy [28]. It is possible that FoxO1 regulates telomerase activity through unknown mechanisms. Therefore, it is extremely important to investigate telomere-associated proteins that might contribute to the pathogenesis of cardiovascular disease.

The present results showed that CR suppressed oxidative stress, based on the measurement of 8-OHdG levels. Furthermore, CR decreased the level of p-Akt and increased the level of eNOS and Sirt1. These effects of CR were abrogated in FoxO1^+/−^ mice. Thus, it is likely that FoxO1 is an important signal protein for maintaining not only telomere biology, but also anti-oxidant activity. The FoxO protein family regulates diverse cellular functions in many cell types, including proliferation, apoptosis, DNA repair, defense against oxidative stress, and autophagy (depending on the cellular environment) [24, 29]. FoxO1 has been assumed to promote nuclear localization of target genes involved in p-Akt signaling [30]. Thus, the FoxO1 gene would have affected WT mice subjected to CR in the present study. However, FoxO1^+/−^ mice did not exhibit any abnormalities of the intracellular signals related to cardiac protection or autophagy-related genes. In fact, the activity of the FoxO1 protein is subject to posttranslational modifications, including phosphorylation, acetylating, and ubiquitylation [29, 31]. We consider that CR may have increased the FoxO1 protein levels in heart tissue, inducing oxidative stress-associated phosphorylation of FoxO1, and then might have promoted the translocation of FoxO1 into the nucleus and activated the transcription of FoxO1 target genes.

We observed that CR significantly reduced the LVEDD and LVESD in WT-CR mice but FS was unaltered. These observations were not seen in FoxO1^+/−^-CR. The present results are not considered to have contributed to changes in the body weight. Since the enhanced autophagy might be associated with changes of cardiac geometry during CR [32, 33], it is possible that the LV morphological changes contributed to the induction of anti-oxidant and anti-apoptotic genes. We evaluated the diastolic function by measuring the inflow velocity and the deceleration time of the mitral valve. These diastolic parameters were not altered in any of the experimental mice. It might be contributed to the insufficient duration of CR to assess the diastolic function in this study [3]. In any case, the exact mechanism by which enhanced autophagy preserves LV diastolic function remains to be resolved.

In conclusion, the present study demonstrated that CR increased telomerase activity and autophagy with changing the cardiac geometry and also attenuated oxidative stress in control WT mice. It was also suggested that FoxO1-p-Akt signaling might play a pivotal role in reducing intracellular reactive oxygen species formation [24, 30]. These observations were not seen in mice with partial loss of the FoxO1 gene. Finally, we can point out that FoxO1 signaling may play an important mediator in the response to CR for the metabolic equilibrium. More research will be needed to understand the role of these transcription factors in regulating cardiovascular development, function, and disease as well as to elucidate how FoxO transcription factors interact with the network in cardiovascular disease.
